# FRIENDSHIP IN APHASIA

**DOI:** 10.1093/geroni/igac059.142

**Published:** 2022-12-20

**Authors:** Natalie Douglas

**Affiliations:** Central Michigan University, Mount Pleasant, Michigan, United States

## Abstract

People living with aphasia (PLWA) have identified friendship as a key area of need, and any programs or interventions designed to support this priority should have the input of PLWA at the forefront. This portion of the symposium will outline steps that were taken to engage PLWA in the development of evidence-informed interventions and programming to support friendship for PLWA, despite the challenges PLWA face in communication. The process of developing and using a multi-stakeholder advisory board of clinicians, researchers, PLWA, and family members will be described. Data highlighting key themes from interviews of PLWA, their significant others, and their friends will be presented including reciprocity, persistence, and communication strategies. Finally, communication techniques to engage PLWA in research activities as informed by PLWA themselves, clinicians, and researchers will be provided.

